# Cerebral Ischemia–Reperfusion Injury: Unraveling the Mitophagy–Oxidative Stress Axis for Neuroprotective Strategies

**DOI:** 10.3390/ijms27052448

**Published:** 2026-03-06

**Authors:** Yanling Zhou, Baochun Luo, Tong Shang, Zengrong Wei, Wei Zou

**Affiliations:** 1 Graduate School, Heilongjiang University of Chinese Medicine, Harbin 150040, China; zhouyanl1999@163.com (Y.Z.); 18845126387@163.com (B.L.); shangtong1997@163.com (T.S.); 18830270682@163.com (Z.W.); 2 Heilongjiang University of Chinese Medicine, Harbin 150040, China

**Keywords:** cerebral ischemia–reperfusion injury, ischemic stroke, mitophagy, oxidative stress, signaling pathway

## Abstract

Cerebral ischemia–reperfusion (I/R) injury is a major pathological contributor to neurological deterioration following ischemic stroke (IS) and remains a critical barrier to effective neuroprotection. Accumulating evidence indicates that cerebral I/R injury is driven not by isolated stress responses but by coordinated and dynamic interactions among multiple cellular pathways. Among these, the bidirectional crosstalk between mitophagy and oxidative stress has emerged as a central regulatory axis. Moderate oxidative stress can function as an adaptive signal, activating protective mitophagy through key pathways such as AMPK/ULK1 signaling and cardiolipin externalization, thereby facilitating mitochondrial quality control and maintaining cellular homeostasis. Conversely, appropriately regulated mitophagy limits excessive reactive oxygen species (ROS) production by removing dysfunctional mitochondria, forming a negative feedback mechanism. However, dysregulation or excessive activation of either process disrupts this balance, leading to a self-amplifying cycle of mitochondrial dysfunction and oxidative damage that exacerbates neuronal injury. This review systematically summarizes the molecular mechanisms governing the oxidative stress–mitophagy crosstalk in cerebral I/R injury, highlighting key signaling nodes and regulatory pathways that determine protective versus detrimental outcomes. Furthermore, we discuss emerging therapeutic strategies aimed at precisely modulating this axis in a spatiotemporal- and intensity-dependent manner. By integrating mechanistic insights with translational perspectives, this review provides a conceptual framework for developing targeted neuroprotective interventions based on coordinated regulation of mitochondrial quality control and redox homeostasis.

## 1. Introduction

Stroke is an acute cerebrovascular disease characterized by the sudden rupture or blockage of blood vessels in the brain, leading to insufficient blood supply, severe hypoxia, necrosis, and functional impairment of brain tissue. As a major public health concern, stroke affects approximately 795,000 individuals annually. According to the 2021 Global Burden of Disease (GBD) report, China has the highest incidence of stroke worldwide, reaching 226.4 cases per 100,000 people [1]. A report on the burden of stroke in China indicated a 104% increase in the number of stroke cases from 1990 to 2021 [2]. Based on the degree of cerebral blood flow impairment, stroke can be categorized into hemorrhagic and ischemic types. Ischemic stroke has the highest incidence, at 155.7 cases per 100,000 people, accounting for 87% of all stroke cases [3,4]. Current primary treatments for acute ischemic stroke include intravenous thrombolysis, antiplatelet therapy, and endovascular thrombectomy. Tissue plasminogen activator (t-PA) is the only pharmacological treatment approved by the U.S. Food and Drug Administration (FDA) for ischemic stroke. However, t-PA therapy is limited by a narrow time window (<4.5 h), beyond which the risk of hemorrhagic transformation (HT) increases [5]. Additionally, reperfusion following thrombolysis may lead to secondary neuronal injury [6]. Mitochondria are increasingly recognized as the central hub integrating bioenergetic depletion, redox imbalance, calcium overload, and innate immune activation during cerebral ischemia–reperfusion (I/R) injury [7]. During ischemia, the abrupt cessation of oxygen and glucose supply rapidly disrupts oxidative phosphorylation, leading to ATP depletion and ion homeostasis disruption. During reperfusion, reactivation of the electron transport chain may trigger an explosion of mitochondrial reactive oxygen species (mtROS), exacerbating oxidative damage to lipids, proteins, and nucleic acids. Concurrently, mitochondrial Ca^2+^ overload promotes the opening of the mitochondrial permeability transition pore (mPTP), collapse of the mitochondrial membrane potential, and release of pro-apoptotic factors, collectively driving apoptosis and necrosis-related cell death pathways. Notably, damaged mitochondria also serve as sources of damage-associated molecular patterns (DAMPs), including mitochondrial DNA (mtDNA), thereby fueling neuroinflammation and exacerbating secondary injury. Therefore, the mitochondrial quality control (MQC) system—encompassing mitochondrial dynamics and selective clearance mechanisms—provides a mechanistic basis for targeting mitophagy as a specific therapeutic target within the cerebral I/R cascade [8,9].

Ischemia/reperfusion injury triggers a complex and temporally evolving cascade involving excitotoxicity, mitochondrial dysfunction, oxidative stress, neuroinflammation, and multiple cell-death pathways, among which mitochondrial impairment and mtROS overproduction represent key drivers linking upstream metabolic stress to downstream neuronal demise [7]. The interaction between oxidative stress and mitophagy is particularly critical. As the primary site of cellular respiration, mitochondria serve as the major source of reactive oxygen species (ROS) [10]. Mitophagy, as a selective form of autophagy, alleviates downstream effects of oxidative stress by clearing damaged mitochondria, thereby reducing excessive ROS production post-ischemia [11]. Conversely, oxidative stress itself modulates mitophagy by activating key signaling pathways (including AMPK/ULK1, cardiolipin, MAPK, and Nrf2 pathways). This establishes a finely tuned bidirectional regulatory network between mitophagy and oxidative stress.

Previous studies have demonstrated that mitophagy and oxidative stress are essential in the pathophysiology of cerebral I/R injury [12,13]; however, existing research reveals a paradoxical phenomenon: this cross-regulation can exert neuroprotective effects while also potentially causing damage. The specific outcomes may depend on factors such as the timing and intensity of activation. Specifically, it remains unclear whether these mechanisms activate or inhibit mitophagy and oxidative stress, and existing literature has not sufficiently elucidated the time-dependent and intensity-dependent nature of these processes. This highlights a significant knowledge gap: how do different signaling pathways differentially interact with oxidative stress and mitophagy following ischemia, and how does this interaction influence neuronal survival? Emerging research evidence also highlights potential therapeutic targets within this interplay [14]. Therefore, this review not only aims to summarize existing knowledge but also strives to integrate comparative research findings and propose potential mechanisms regulating the interaction between oxidative stress and mitophagy in cerebral I/R injury. By addressing these critical questions, we hope to provide new insights for ischemic stroke treatment, focusing on how targeted interventions in these pathways can mitigate neuronal damage and improve clinical outcomes.

## 2. The Generation and Key Regulatory Mechanisms of Mitophagy After Cerebral I/R

In eukaryotic cells, mitochondria are double-membraned organelles primarily responsible for oxidative metabolism, generating ATP essential for various cellular activities, and are thus referred to as the “powerhouses of the cell”. Under conditions of severe oxidative stress, the accumulation of ROS leads to mitochondrial depolarization, activating autophagic signals and inducing the selective degradation of damaged mitochondria, a process known as mitophagy [15]. As a crucial mechanism for maintaining mitochondrial quality control, mitophagy plays an important role in cerebral I/R injury.

Research has shown that following cerebral I/R injury, mitophagy occurs through two main pathways: the ubiquitin-dependent pathway and the non-ubiquitin-dependent/receptor-dependent pathway. The PINK1/Parkin signaling pathway is central to the ubiquitin-dependent mechanism. In contrast, the receptor-dependent pathway typically involves specific receptor proteins (such as BNIP3, NIX/BNIP3L, and FUNDC1) that selectively recognize and mark damaged or dysfunctional mitochondria, ultimately leading to their degradation to preserve normal mitochondrial function [16]. In the following sections, we will focus on these two mitophagy mechanisms.

### 2.1. Ubiquitin-Dependent Pathway

PINK1/Parkin is regarded as the dominant pathway mediating mitophagy and is also the most extensively studied ubiquitin-dependent pathway. Following cerebral ischemia, the PINK1/Parkin signaling cascade is activated in microglia in response to acute damage such as loss of mitochondrial membrane potential [17,18]. This process initiates with the stabilization of PTEN-induced kinase 1 (PINK1), a nuclear-encoded serine/threonine kinase that functions as a sensor of mitochondrial health [19].

In healthy mitochondria, PINK1 is continuously imported into the inner mitochondrial membrane and rapidly degraded. However, upon loss of mitochondrial membrane potential, PINK1 accumulates on the outer mitochondrial membrane (OMM) and interacts with the translocase of the outer membrane (TOM) complex, which serves as an activation platform [20]. Subsequently, activated PINK1 recruits the cytosolic E3 ubiquitin ligase Parkin to the OMM. Parkin mediates the ubiquitination of multiple OMM proteins, resulting in the formation of polyubiquitin chains. These Parkin-generated polyubiquitin chains are crucial signals for initiating downstream autophagosome formation [21,22].

Upon binding of autophagy receptors like p62/SQSTM1 and NBR1 to ubiquitinated damaged mitochondria, they interact with microtubule-associated protein 1 light chain 3 (LC3) or its homologues in the GABARAP family, via their LC3-interacting region (LIR), which are present on the phagophore [23,24,25]. This interaction effectively “anchors” the ubiquitinated damaged mitochondria to the nascent autophagosomal membrane. As the phagophore elongates and engulfs the mitochondrion, a double-membrane autophagosome forms, completely encapsulating the damaged organelle.

Subsequently, the autophagosome matures and fuses with a lysosome, forming an autolysosome. Within the autolysosome, the damaged mitochondrion is thoroughly degraded by lysosomal enzymes, such as hydrolases, and its components are recycled [26]. This process completes the selective autophagy of damaged mitochondria, maintaining mitochondrial health and functional homeostasis within the cell.

Recent evidence has further demonstrated that the homeostatic balance and activation capacity of PINK1 are tightly regulated by the Heat shock protein 90 (Hsp90) chaperone system. Notably, studies have directly demonstrated that Hsp90 forms a unique complex with PINK1 and chaperones such as Cdc37 and FKBP51. This complex supports PINK1 stability, thereby affecting the selective clearance of damaged mitochondria [27,28]. Simultaneously, modulation of Hsp90 activity alters PINK1 levels and influences mitophagy; for example, inhibition of mitochondrial Hsp90 with targeted agents such as Gamitrinib-TPP induces PINK1 accumulation, promotes Parkin recruitment to mitochondria, and activates downstream mitophagic signaling, indicating that the chaperone status of PINK1 directly affects mitophagy initiation [28]. The involvement of Hsp90 in PINK1/Parkin-mediated mitophagy introduces a promising therapeutic dimension for cerebral I/R injury. Given that Hsp90 inhibitors can modulate mitophagy flux and confer neuroprotection in preclinical stroke models [29], targeting Hsp90-PINK1 interactions may offer a strategy to fine-tune mitochondrial quality control.

### 2.2. Ubiquitin-Independent Mitophagy (Receptor-Dependent)

#### 2.2.1. BNIP3 and BNIP3L/NIX Pathway

BNIP3 (Bcl-2/adenovirus E1B 19 kDa interacting protein 3) and its homolog BNIP3L (also known as NIX) are Bcl-2 family members localized to the outer mitochondrial membrane. Initially characterized as pro-apoptotic factors, they were later implicated in mitochondrial clearance during reticulocyte maturation [30,31]. Mitophagy mediated by the BNIP3 and BNIP3L/NIX pathway primarily acts on neuronal cells, particularly cortical neurons [32]. Recent studies have identified them as key mitophagy receptors in cerebral I/R injury, critically mediating the removal of damaged mitochondria [33]. Hypoxia and excitotoxic stress following cerebral ischemia activate hypoxia-inducible factor-1α (HIF-1α) and forkhead box transcription factor O3 (FOXO3), which directly bind to the promoter regions of the BNIP3 and NIX genes [33,34]. This binding significantly upregulates their transcription and protein expression, providing a molecular basis for mitophagy initiation [35]. The function of OMM-localized BNIP3 and NIX is heavily regulated by post-translational modifications, particularly phosphorylation [36]. Phosphorylation of NIX at Ser34 and Ser35, proximal to its N-terminal LC3-interacting region LIR, enhances its affinity for LC3 and GABARAP family proteins by approximately two orders of magnitude [37]. Similarly, BNIP3 requires phosphorylation at Ser17, located near its LIR domain, for efficient recruitment of LC3 or GATE-16 (ATG8 family members) [38]. Via their phosphorylation-activated LIR motifs, BNIP3/NIX directly act as autophagy receptors, anchoring the phagophore to damaged mitochondria and initiating autophagosome formation [39]. Beyond this direct receptor function, they synergistically promote autophagy through indirect mechanisms. BNIP3 and NIX compete with the anti-apoptotic protein Bcl-2 for Beclin-1 binding via their BH3 domain, disrupting the Bcl-2/Beclin-1 complex. The subsequent release of Beclin-1 promotes autophagy initiation complex formation, thereby activating mitophagy [40]. Furthermore, BNIP3L can function downstream of the PINK1/Parkin pathway, enhancing mitophagy via PARK2-mediated ubiquitination. This process facilitates the mitochondrial localization of the adaptor protein NBR1, which binds LC3 and ultimately triggers mitophagy [41].

Through these synergistic, multi-faceted mechanisms, BNIP3- and NIX-mediated mitophagy is effectively activated, enabling the timely removal of dysfunctional mitochondria generated following cerebral ischemia. This process markedly reduces the release of pro-apoptotic factors, such as cytochrome c, inhibiting caspase-dependent apoptotic cascades [42]. Concurrently, it diminishes mitochondrial-derived ROS levels, attenuating oxidative stress injury [43]. Consequently, this pathway maintains mitochondrial quality control following cerebral ischemia, reduces neuronal death, and exhibits significant neuroprotective functions.

#### 2.2.2. FUNDC1 Pathway

FUNDC1 is a transmembrane protein localized to the outer mitochondrial membrane, mediating hypoxia-induced mitophagy in mammals. The activation of FUNDC1-mediated mitophagy primarily occurs in neurons, and FUNDC1 is also highly expressed in microglia [44]. Its structure comprises three transmembrane domains, with the N-terminal domain exposed to the cytoplasm and containing an LIR, while the C-terminus extending into the intermembrane space of mitochondria [45]. The function of FUNDC1 is primarily regulated by its phosphorylation status. Under normal physiological conditions, Src kinase phosphorylates Tyr18, and casein kinase 2 (CK2) phosphorylates Ser13, inhibiting the interaction between FUNDC1 and LC3, thereby suppressing mitophagy. Conversely, under hypoxic or mitochondrial stress conditions, the activities of Src and CK2 are suppressed, while phosphatase PGAM5 dephosphorylates Ser13, and kinase ULK1 phosphorylates Ser17 [46,47,48]. These modifications collectively enhance the affinity of FUNDC1 for the autophagy key protein LC3, promoting the initiation of mitophagy [49]. Furthermore, FUNDC1 promotes mitochondrial fission, thereby further facilitating autophagic clearance, by interacting with dynamin-related protein 1 (Drp1) and modulating its interaction with optic atrophy 1 (OPA1) [50,51]. In ischemic diseases, the stability of FUNDC1 is generally regulated by ubiquitination; for example, it has been reported in ischemic heart disease that E3 ligase MARCH5 mediates ubiquitination of FUNDC1 at K119 during the early stages of hypoxia, leading to its degradation via the proteasome pathway [52,53]. This negative feedback mechanism prevents excessive autophagy, providing cells with a buffer time to respond to stress, and similar regulation may exist in cerebral ischemia. In recent years, FUNDC1 has received increasing attention, and studies have demonstrated that activating FUNDC1-mediated mitophagy in the post-ischemic phase of ischemic stroke may serve as a potential therapeutic strategy for ischemic stroke [44].

The three pathways collectively underscore the cellular response to oxidative stress in cerebral I/R injury, with each playing a vital role in mitochondrial quality control. While the PINK1/Parkin pathway is primarily focused on sensing and responding to acute mitochondrial damage, the BNIP3 and FUNDC1 pathways are more attuned to the gradual adaptation to hypoxic conditions, ensuring that damaged mitochondria are timely removed before they can contribute to cellular dysfunction. It is noteworthy that these pathways are not entirely independent but exhibit cross-talk. For instance, BNIP3L/NIX has also been reported to act as a downstream effector molecule in the PINK1/Parkin pathway, enhancing mitophagy [54].

In the context of cerebral I/R injury, however, it is important to emphasize that mitophagy is not invariably beneficial. While the clearance of damaged mitochondria leads to a reduction in ROS and contributes to cellular protection, excessive activation of the PINK1/Parkin pathway, BNIP3 pathway and FUNDC1 pathway can result in energy depletion and may compete with apoptosis pathways for cellular resources and even aggravated overall pathological consequences of cerebral I/R injury [55,56]. This duality underscores the necessity for balanced responses during ischemic events, making the dynamic crosstalk equilibrium between mitophagy and oxidative stress particularly crucial.

Importantly, growing evidence indicates that the regulation of mitophagy and its functional consequences in cerebral I/R injury exhibit high cell-type specificity. Although core signaling pathways, such as PINK1/Parkin, BNIP3/NIX, and FUNDC1, share commonalities across different cell populations, their upstream triggering mechanisms and downstream effects show significant differences among distinct components of the neurovascular unit.

Mitophagy in neurons is primarily triggered by energy depletion, Ca^2+^ overload, and ROS accumulation. Typical upstream regulatory axes include the PINK1/Parkin pathway and hypoxia-associated BNIP3/NIX pathways. Moderately activated mitophagy aids in clearing damaged mitochondria, reducing oxidative stress levels, and mitigating apoptosis. However, excessive or sustained activation may further exacerbate ATP depletion, thereby increasing neuronal death risk and exhibiting distinct double-edged sword characteristics [9,57].

In contrast, astrocytes exhibit a more metabolically adaptive response. Mitophagy in astrocytes is closely associated with hypoxia-responsive and metabolic stress signaling pathways and primarily functions to maintain mitochondrial homeostasis and redox balance [58]. By preserving metabolic stability, astrocytic mitophagy indirectly supports neuronal survival and contributes to microenvironmental regulation following reperfusion [59].

In microglia, mitophagy is tightly linked to inflammatory modulation. The accumulation of mtROS and mtDNA can potentiate inflammasome activation, with damaged mitochondria acting as an amplifier of pro-inflammatory signals [60]. Conversely, PINK1/Parkin dependent mitophagy restrains NLRP3 inflammasome signaling and the release of pro-inflammatory cytokines [61]. Thus, in this context, mitophagy primarily regulates neuroinflammation rather than directly determining cell survival.

At the level of the neurovascular unit, mitophagy in brain microvascular endothelial cells (BMECs) responds predominantly to oxidative and metabolic stress, contributing to the maintenance of tight junction integrity and blood–brain barrier (BBB) stability [62,63]. Dysregulated mitochondrial quality control in endothelial cells may aggravate barrier permeability and edema formation. Emerging evidence further suggests that mitochondrial homeostasis in pericytes may influence microvascular tone and post ischemic vascular remodeling [64].

Collectively, these findings indicate that mitophagy in cerebral I/R injury cannot be regarded as a uniform protective or detrimental mechanism. Instead, it exerts distinct functional roles depending on the cellular context, modulating survival thresholds in neurons, inflammatory activation in microglia, metabolic support in astrocytes, and vascular integrity within the neurovascular unit. This cell-type specificity underscores the necessity of precisely timed and targeted therapeutic strategies when modulating mitophagy in ischemic stroke.

The cell type specific characteristics of mitophagy in cerebral I/R injury are summarized in Table 1.

## 3. Generation and Regulatory Mechanisms of Oxidative Stress Following Cerebral I/R

### 3.1. Physiological Basis of Oxidative Stress Production

The brain’s inherent high metabolic rate and lipid enrichment render it highly susceptible to oxidative damage [65].

During cerebral I/R injury, mitochondrial dysfunction leads to excessive intracellular ROS and reactive nitrogen species (RNS) generation. The rate of production surpasses the capacity of the endogenous antioxidant system, disrupting the redox balance and inducing oxidative stress. This imbalance results in ROS/RNS-mediated damage to critical biomolecules, including lipids, proteins, and DNA, ultimately triggering cell apoptosis or necrosis [66]. While ROS function as important intracellular signaling molecules under physiological conditions, their excessive accumulation during cerebral I/R significantly exacerbates structural and functional damage to brain tissue and plays a central role in the pathogenesis of ischemic stroke through complex molecular networks. Importantly, beyond their pathogenic effects, elevated ROS also act as critical signaling molecules that initiate and regulate mitophagy. The following sections will focus on elucidating the mechanisms of ROS generation in this context.

### 3.2. The Main Sources of ROS

As a byproduct of oxidative phosphorylation, electron leakage in the mitochondrial electron transport chain (ETC) generates superoxide. Early studies estimated that approximately 1–2% of oxygen consumed during physiological respiration is utilized to generate mitochondrial reactive oxygen species (mROS) [67]. Although this proportion varies across cell types and energy demands, and exhibits lower transient bursts than NADPH oxidase, mitochondria remain a key intracellular ROS source due to their ubiquity and continuous activity in most eukaryotic cells.

However, the role of ROS is dual; it serves as a signaling molecule in normal physiological processes but can also contribute to cellular damage during pathological conditions, such as the ischemic phase of stroke, the interruption of glucose and oxygen supply leads to increased anaerobic respiration and the accumulation of lactate, causing metabolic acidosis [68]. This highlights the need to underscore the “double-edged sword effect” of ROS in cerebral I/R injury. ETC dysfunction halts ATP synthesis, leading to energy depletion [69]. Respiratory chain blockage also reduces mitochondrial membrane potential, promoting intracellular accumulation of calcium and sodium, and inducing cytotoxic edema. Calcium overload triggers the opening of the mPTP, leading to complete collapse of mitochondrial membrane potential (ΔΨm), creating a vicious cycle. This process releases cytochrome c and activates apoptosis executioner proteins such as caspase-3, ultimately initiating programmed cell death [70]. During reperfusion, the reintroduction of oxygen to the tissue, coupled with the incomplete recovery of ETC function, causes electron leakage from respiratory chain complexes (particularly complexes I and III). These leaked electrons combine with oxygen to generate a burst of superoxide anions (O_2_^−^·), which then initiates a chain reaction producing hydroxyl radicals (·OH) and hydrogen peroxide (H_2_O_2_), among other ROS [71]. This “oxidative storm” not only directly damages biomolecules such as lipids, proteins, and DNA but also synergizes with calcium overload to promote sustained mPTP opening, forming a self-perpetuating cycle that significantly exacerbates neuronal injury [72,73]. While oxygen supply allows mitochondria to resume activity, it also leads to the explosive generation of ROS due to the damaged electron transport chain [74].

**Figure 1:**
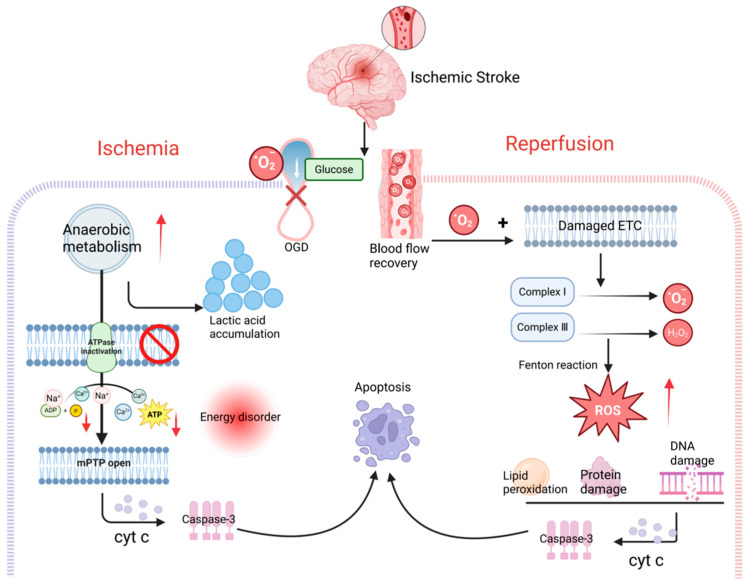
This diagram summarizes the key pathological events. During ischemia, oxygen/glucose deprivation (OGD) shifts metabolism to anaerobic glycolysis causing lactate accumulation and energy failure, leading to calcium overload and mPTP opening which triggers cytochrome c release and apoptosis. During reperfusion, oxygen reintroduction to the damaged electron transport chain results in massive ROS burst from complexes I and III. The resulting oxidative storm via processes including the Fenton reaction directly damages cellular components and synergizes with calcium dysregulation to perpetuate a vicious cycle of mPTP opening and cell death. In the figure, Red “×”: Represents the interruption/blockade of a biological process or supply (e.g., halt of oxygen/glucose supply during oxygen-glucose deprivation (OGD); inactivation of ATPase activity). Upward arrow (↑): Represents the increase in the level/intensity of a substance or process (e.g., enhancement of anaerobic metabolism, accumulation of lactic acid, burst of ROS). Downward arrow (↓): Represents the decrease in the level/intensity of a substance or process (e.g., reduction in ATP content). Created in BioRender. yanling, Z. (2026) https://BioRender.com/zt9gp63.

Collectively, the effects of ROS are highly dependent on their levels, duration, and the stage of ischemic stroke. Low levels of transient ROS serve as crucial signaling molecules, participating in the activation of adaptive protective mechanisms such as pre-conditioning. Conversely, high levels of sustained ROS—particularly those generated in large quantities from multiple sources during the early phase of reperfusion—primarily exert toxic damaging effects [75]. Recognizing this duality also provides a theoretical basis for processes like mitophagy potentially exhibiting both protective and harmful properties (Figure 1).

#### 3.2.1. ROS Production by Xanthine Oxidase

In addition to mitochondria, xanthine oxidase (XO) and nicotinamide adenine dinucleotide phosphate oxidase (NOX) are two major key enzyme systems mediating the explosive ROS production during the early phase of reperfusion [76].

Under physiological conditions, xanthine oxidoreductase (XOR) predominantly exists as xanthine dehydrogenase (XDH). However, during the ischemic phase, severe energy depletion leads to the breakdown of ATP and the subsequent intracellular accumulation of hypoxanthine. Concurrently, XDH is proteolytically converted into XO, providing the substrate and catalyst for ROS bursts. Although this “priming” occurs during ischemia, the explosive generation of ROS is triggered only upon reperfusion. When oxygen is restored, XO catalyzes reactions at its maximum rate, utilizing hypoxanthine as a substrate, ultimately leading to a burst of ROS [77,78].

#### 3.2.2. ROS Production by NADPH Oxidases

NADPH Oxidases (NOX) represent another significant, regulated source of ROS during cerebral I/R injury, and exert distinct pathophysiological effects at different stages of reperfusion. Early reperfusion phase (minutes to hours), glucose restoration is a critical prerequisite for NOX activation and subsequent massive ROS generation [79]. The catalytic core of NOX (primarily the gp91phox/Nox2 subunit) utilizes NADPH as an electron donor, transferring electrons across the membrane to oxygen molecules to directly generate O_2_^−^·. This process subsequently produces reactive oxygen species such as hydrogen peroxide (H_2_O_2_), significantly intensifying the early oxidative burst [80,81]. Late phase of reperfusion (hours to days), NOX is persistently or reactivated in multiple cell types, including endothelial cells, activated microglia/macrophages, and infiltrating neutrophils. At this stage, NOX not only continuously generates ROS but also interacts with key signaling pathways such as NF-κB and NLRP3 inflammasome, forming a persistent positive feedback loop of inflammation and oxidative stress [82]. This process directly compromises the integrity of the blood–brain barrier and impairs neurovascular unit function by continuously exacerbating oxidative stress, ultimately prolonging and aggravating neurological damage.

#### 3.2.3. ROS/RNS Production by Nitric Oxide Synthase (NOS)

In addition to mitochondria, XO and NOX, nitric oxide synthase (NOS) represents a critical enzymatic source of reactive nitrogen species (RNS) during cerebral I/R injury. It comprises three isoforms: neuronal nNOS, endothelial eNOS, and inducible iNOS. These isoforms exhibit differential regulation during the various phases of ischemia and reperfusion. Under physiological conditions, NO produced by eNOS contributes to the maintenance of cerebral vascular tone and perfusion, exerting a certain vascular protective effect. In contrast, during ischemia and the early reperfusion phase, excitotoxicity and Ca^2+^ overload can rapidly activate nNOS, leading to an acute elevation of NO. In the mid-to-late stages of reperfusion, inflammatory signals drive the sustained expression of iNOS in microglia, astrocytes, and endothelial cells. The calcium-independent, high-output generation of NO by iNOS further amplifies nitrosative stress [5,83].

Additionally, under oxidative stress conditions, the oxidation of BH_4_ or relative deficiency of L-arginine can induce decoupling of NOS, shifting its function from NO production to O_2_^−^· generation. This subsequently interacts with ROS originating from mitochondria, XO, and NOX, promoting the synergistic accumulation of ROS and RNS, thereby exacerbating reperfusion injury [84].

In summary, oxidative stress during cerebral I/R is not the result of a single mechanism, but rather a “vicious cycle” network formed by mitochondrial dysfunction, cascade activation of multiple oxidases (XO, NOX), and dysregulation of the NOS system. A profound understanding of this complex, multi-source regulatory mechanism is crucial for developing targeted antioxidant therapeutic strategies that address specific sources or specific pathological stages.

### 3.3. Antioxidant Stress Response Pathways

In cerebral I/R injury, the overproduction of ROS is a pivotal factor triggering oxidative stress. To counteract this stress, endogenous antioxidant defense systems are activated. These systems primarily consist of two categories: the enzymatic antioxidant system, including SOD, CAT, glutathione peroxidase (GPx), and glutathione reductase (GR); and the non-enzymatic antioxidant system, which comprises glutathione, vitamin C, vitamin E, and coenzyme Q10 (CoQ10), among others [80]. Furthermore, the upregulation and activation of these antioxidant systems are precisely regulated by multiple signaling pathways.

#### 3.3.1. Keap1/Nrf2/ARE Pathway

The Kelch-like ECH-associated protein 1 (Keap1)/nuclear factor erythroid 2-related factor 2 (Nrf2)/antioxidant response element (ARE) signaling pathway is recognized as a crucial antioxidant defense mechanism [85]. Nrf2 is a key intracellular transcription factor that regulates antioxidant stress responses, primarily activating antioxidant responses and promoting the production of antioxidant proteins and enzymes [86]. Under normal physiological conditions, the majority of Nrf2 is sequestered in the cytoplasm by inhibitory protein Keap1, preventing unnecessary activation of downstream antioxidant genes [87].

Upon exposure to excessive ROS or under conditions of oxidative stress, this pathway is rapidly activated [88]. Critical cysteine residues (active thiol groups) on Keap1 are attacked by ROS, leading to conformational changes that impair Keap1’s ability to effectively bind Nrf2 or promote its ubiquitination [89]. The synthesis of Nrf2 is stabilized and increased, and the accumulated Nrf2 translocates from the cytoplasm to the nucleus. In the nucleus, Nrf2 forms a heterodimer with sMaf proteins. This complex specifically recognizes and binds to AREs, initiating the transcription and expression of a series of genes encoding antioxidant proteins, including heme oxygenase-1 (HO-1), glutathione S-transferase (GST), SOD, and CAT [90,91,92].

HO-1 is considered an inducible enzyme, and its antioxidant mechanism involves the degradation of the pro-oxidant molecule heme into iron ions, carbon monoxide (CO), and biliverdin. Biliverdin is subsequently reduced to bilirubin by biliverdin reductase, and bilirubin is a potent endogenous antioxidant [93,94]. Furthermore, the released iron ions bind to transferrin, helping to maintain iron homeostasis and reducing oxidative damage caused by Fenton reactions [95]. The GST family catalyzes the conjugation of glutathione (GSH) to electrophilic compounds, eliminating cytotoxic substances from the body and protecting cells [96,97]. Recent reports indicate that SOD and CAT can scavenge excess O_2_^−^· and H_2_O_2_, respectively, protecting cells from free radical-induced oxidative stress [98].

Although these antioxidant enzymes employ different mechanisms to achieve antioxidation, they do not act in isolation. Instead, they function within a sophisticated antioxidant network, comprehensively enhancing cellular antioxidant, detoxification, and repair capabilities, thereby protecting cells from oxidative stress damage.

Notably, this pathway not only constitutes the core of cellular antioxidant defense, but its activation may also extensively intersect with other key mechanisms maintaining cellular homeostasis, particularly mitochondrial quality control processes. Research indicates that Nrf2 signaling plays a potential regulatory role in maintaining mitochondrial functional integrity and modulating mitophagy. Through its extensive transcriptional regulatory network, Nrf2 may indirectly influence the initiation and progression of mitophagy. This enables it to synergistically maintain mitochondrial network equilibrium and cellular metabolic homeostasis under oxidative stress conditions. Such potential synergistic interactions with pathways like mitophagy further expand the global role of the Keap1/Nrf2/ARE pathway in cellular integrated stress responses and survival protection.

#### 3.3.2. HIF-1 Pathway

It is well established that hypoxia-inducible factor 1 (HIF-1), an oxygen-sensitive transcription factor composed of HIF-1α and HIF-1β subunits, serves as a key regulator of cellular adaptation. By modulating the expression of over 700 target genes, HIF-1 plays a central role in antioxidant responses [99,100]. Although the precise mechanisms remain incompletely elucidated, they may involve promoting antioxidant enzyme expression, reprogramming energy metabolism, inducing cytoprotective factors, and suppressing cell death, thereby widely participating in various pathological and adaptive processes [101,102,103].

Under normoxic conditions, prolyl hydroxylase domain (PHD) proteins hydroxylate HIF-1α, which is then recognized by the von Hippel-Lindau tumor suppressor protein (pVHL) complex, leading to ubiquitination and subsequent rapid degradation via the proteasome pathway [104,105]. Under hypoxic conditions, the hydroxylation activity of PHD is inhibited due to the lack of molecular oxygen, resulting in the accumulation and stabilization of HIF-1α in the cytoplasm. The stabilized HIF-1α translocates into the nucleus, dimerizes with HIF-1β, and forms a transcriptionally active heterodimer [106]. This complex binds to hypoxia-response elements (HREs) in the promoter regions of target genes, initiating the transcription of multiple genes, including erythropoietin (EPO), vascular endothelial growth factor (VEGF), glucose transporter 1 (GLUT1), superoxide dismutase 1 (SOD1), and heme oxygenase-1 (HO-1) [107,108,109].

These downstream effectors exert distinct antioxidant functions. For instance, Zhang, et al. (2016) [110] demonstrated that VEGF not only promotes angiogenesis and improves oxygen supply to ischemic tissues, thereby alleviating oxidative damage; moreover recent studies also suggest that VEGF-B possesses intrinsic antioxidant properties [111]. HO-1 exerts its antioxidant effects primarily by degrading toxic heme into CO and biliverdin/bilirubin. Moreover, experimental evidence indicates that suppression of GLUT1 leads to elevated ROS levels, underscoring its important role in cellular adaptation to oxidative stress [112].

However, it is important to note that the biological effects of HIF-1 are highly context-dependent and may exhibit more complex, even biphasic, modes of action under specific pathological conditions. For example, Chen, et al. [113] found that silencing HIF-1α reduced ROS generation to some extent via the CXCR4/NF-κB pathway, whereas Cheng, et al. [114] reported that HIF-1 could promote neuronal death after ischemic stroke. Further studies have revealed a time-dependent duality in HIF-1α function: during the early ischemic phase (approximately 1~12 h), rapid accumulation of HIF-1α primarily activates pro-apoptotic genes (e.g., Bax) and pro-inflammatory factors (e.g., TNF-α) via binding to HREs in their promoters, exacerbating neuronal death. In contrast, during the late ischemic phase (beyond 48 h), sustained HIF-1α expression upregulates protective factors such as EPO and VEGF, promoting neuronal survival, angiogenesis, and tissue repair, thereby contributing to neurological recovery [115]. Importantly, HIF-1α serves as a bridge in the cross-regulation between oxidative stress and mitophagy. Under hypoxic conditions, HIF-1α transcritally activates genes such as BNIP3, NIX, and FUNDC1. These proteins serve not only as key receptors for mitophagy but also participate in regulating mitochondrial ROS production, thereby tightly coupling oxidative stress signaling with mitochondrial quality control mechanisms.

Thus, accumulating evidence demonstrates that HIF-1α plays a time-dependent biphasic role in cerebral I/R injury.

In conclusion, HIF-1α orchestrates a fundamental antioxidant and cytoprotective program under hypoxic conditions. Nevertheless, its role extends beyond a uniformly beneficial response, as evidenced by its dual and opposing functions in specific pathological contexts like cerebral I/R injury. Therefore, the overall impact of HIF-1α is determined by the intricate balance between its pro-survival transcriptional targets and its potential to drive deleterious processes, highlighting that its net effect on oxidative stress and cell fate is both specific and temporally regulated. Specifically, by regulating pathways such as BNIP3, NIX, and FUNDC1, HIF-1α synergistically integrates the oxidative stress response with mitophagy mechanisms, forming a key regulatory network that maintains cellular homeostasis under hypoxic stress.

In summary, oxidative stress represents a central pathological mechanism in cerebral I/R injury. Its generation stems from multifactorial bursts of ROS originating from mitochondrial dysfunction, XO, and NADPH oxidase, and is precisely regulated by key signaling pathways such as Keap1/Nrf2/ARE and HIF-1. Notably, ROS plays a dual-edged role in this process: low-level, transient ROS serve as crucial signaling molecules to activate adaptive protective mechanisms, including mitophagy; whereas high-level, sustained ROS directly cause oxidative damage to lipids, proteins, and DNA, driving apoptosis or necrosis. This highly dynamic and stage-dependent interaction establishes a complex, bidirectional dialogue between oxidative stress and mitophagy: oxidative stress signals initiate mitophagy to clear damaged mitochondria and maintain cellular homeostasis, while the progression of mitophagy in turn influences ROS production and clearance. This interwoven regulatory network forms the foundation for the progression of cerebral I/R injury and endogenous repair, providing a critical logical starting point for subsequent investigations into the crosstalk between oxidative stress and mitophagy.

## 4. The Crosstalk Mechanisms Between Mitophagy and Oxidative Stress

Cerebral I/R injury is a complex process involving multiple signaling pathways. Within this context, oxidative stress, a critical regulator of cellular state, and mitophagy engage in a dynamic, reciprocal interplay [116]. On one hand, a moderate increase in oxidative stress can activate specific redox signaling pathways. ROS, serving as the key signaling molecules of oxidative stress, act as a crucial bridge linking oxidative stress to mitophagy [117]. They can induce the initiation of mitophagy, thereby facilitating the clearance of damaged mitochondria and the maintenance of cellular homeostasis. On the other hand, the timely activation of mitophagy effectively removes the primary source of ROS, establishing a negative feedback loop that helps to suppress the exacerbation of oxidative stress [118,119]. However, a disruption in this balanced relationship can lead to a vicious cycle; persistently high levels of oxidative stress may either excessively induce or suppress mitophagy, while chronic dysregulation of mitophagic function can, in turn, contribute to ROS accumulation [120].

**Figure 2:**
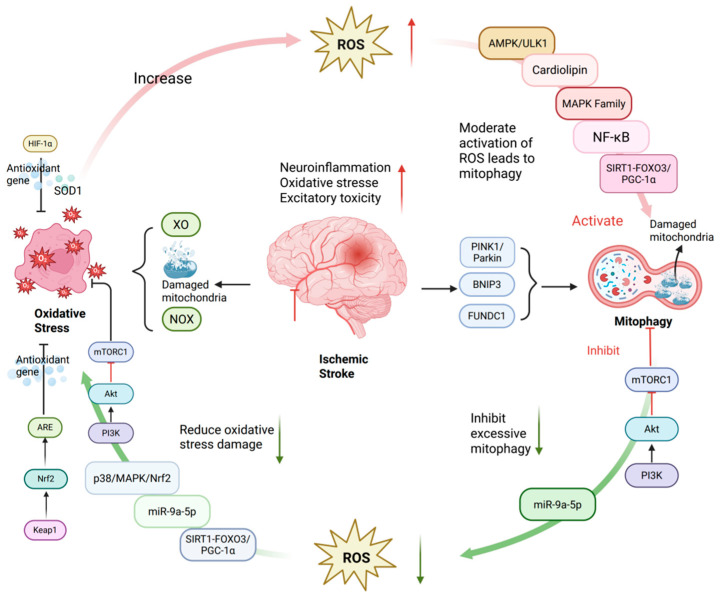
Schematic of bidirectional crosstalk between mitophagy and oxidative stress in ischemic stroke. Key pathways include AMPK/ULK1, Cardiolipin, MAPK, and NF-κB mediating mitophagy activation; PINK1/Parkin, BNIP3, and FUNDC1 facilitating damaged mitochondria clearance; PI3K/Akt/mTOR, miR-9a-5p, and SIRT1-FOXO3/PGC-1α regulating redox balance and mitophagy homeostasis. In the figure, Red upward arrow (↑): Represents the activation of a pathway/process or the increase in the level/intensity of a molecule/biological event (e.g., elevation of ROS, enhancement of neuroinflammation/oxidative stress/excitatory toxicity). Green downward arrow (↓): Represents the inhibition of a pathway/process or the decrease in the level/intensity of a molecule/biological event (e.g., alleviation of oxidative stress damage, suppression of excessive mitophagy). “⊥” (inhibitory arrow): Represents the inhibitory regulatory effect of one molecule/process on another (e.g., HIF-1α inhibits antioxidant genes like SOD1; oxidative stress inhibits ARE). Created in BioRender. yanling, Z. (2026) https://BioRender.com/rlrd2ei.

The key to maintaining this equilibrium lies in a series of precise molecular regulatory mechanisms. Aside from the aforementioned PINK1/Parkin and other pathways, the following signaling pathways also play indispensable roles in mediating the crosstalk between oxidative stress and mitophagy in cerebral I/R injury (Figure 2)

### 4.1. AMPK/ULK1

Oxidative stress/ROS serve as primary inducers of mitophagy, mediating the identification, engulfment, and disposal of compromised mitochondria [121,122]. Accumulating evidence suggests that the AMPK/ULK1 pathway serves as a pivotal link connecting energy crises, oxidative stress, and the initiation of mitophagy. Previous studies have demonstrated that ROS primarily regulate mitophagy through the cytoplasmic mammalian target of rapamycin (mTOR) [121]. It is worth emphasizing that ROS is not only a product of mitochondrial damage but also serves as a key upstream signal for activating AMPK. Under nutrient-replete conditions, mTOR complex 1 (mTORC1) is activated and phosphorylates ULK1 (Unc-51 like autophagy activating kinase 1) and Atg13, thereby suppressing their kinase activity, while AMP-activated protein kinase (AMPK) remains inactive [123]. During cerebral I/R injury, interrupted oxygen and glucose supply leads to hypoxia, nutrient deprivation, and ROS accumulation [124,125,126]. These stressors activate AMPK, which in turn inhibits mTORC1 activity and relieves its suppression of the ULK1 complex. It is widely accepted that the ULK1 complex serves as a key molecular switch for initiating mitophagy [127]. This cascade ultimately enhances phosphorylation of ULK1 and its adaptor protein Atg13, triggering mitophagy to eliminate damaged mitochondria, attenuate further ROS accumulation, and maintain cellular energy homeostasis [128].

Interestingly, AMPK/ULK1-mediated mitophagy exhibits a distinct biphasic effect: moderate activation serves as a crucial cellular protective mechanism, whereas excessive or sustained activation may lead to excessive mitochondrial clearance, insufficient energy supply, and even promote cell death. Therefore, precise regulation of this pathway is key to achieving neuroprotection.

### 4.2. Cardiolipin

Cardiolipin (CL) is a key signaling lipid for oxidative stress-driven mitophagy, primarily localized within the inner mitochondrial membrane (IMM) [129].

Under normal physiological conditions, CL accumulates in the inner membrane and interacts with proteins to maintain mitochondrial morphology and function. However, under excessive ROS stimulation, CL undergoes peroxidation and structural rearrangement, flipping from the inner membrane matrix side to the outer membrane cytoplasmic side. This externalization process is a key step in initiating mitophagy [130].

Experimental studies have demonstrated that prion protein PrP106-126-induced mitophagy depends on the externalization of CL to the outer mitochondrial membrane [131]. Inhibition of this externalization process hinders the clearance of damaged mitochondria, leading to more severe mitochondrial dysfunction and exacerbated oxidative stress. Therefore, CL externalization is a crucial step in initiating mitophagy. CL externalized to the cytosolic side acts as an “eat-me” signal, directly recognized and bound by LC3, a key autophagy protein, thereby promoting autophagosome engulfment of damaged mitochondria, This process constitutes an important form of non-receptor-dependent mitophagy [132]. Subsequently, autophagosomes fuse with lysosomes, and their contents are degraded, initiating a mitophagy pathway that does not rely on specific protein receptors.

Concurrently, the inner membrane protein PHB2 can also function as a mitophagy receptor, binding to LC3 following Parkin-mediated outer membrane rupture, activating mitophagy [133]. Studies indicate that the interaction between PHB2 and CL at the LC3/GABARAP protein level jointly regulates the initiation and execution of mitophagy [134].

In summary, CL serves as a bridge between oxidative damage sensing and mitochondrial clearance, acting as a core molecule in maintaining mitochondrial quality control and cellular homeostasis.

### 4.3. MAPK

The MAPK family comprises a group of highly conserved mitogen-activated protein kinases, with key members including extracellular signal-regulated kinase (ERK), p38 MAPK, and c-JUN N-terminal kinase (JNK) [135]. These pathways exhibit distinct functional focuses: ERK primarily regulates cell proliferation and survival, JNK governs stress-induced apoptosis, while p38 MAPK is closely involved in processes such as inflammation, aging, and autophagy [136,137,138]. In ischemic stroke models, numerous studies have elucidated the expression levels, phosphorylation status, and cellular distribution of MAPKs [139,140,141]. In neuroblastoma research, one reported mechanism is the selective activation of ERK1/2 and JNK MAPK pathways under moderate oxidative stress. This activation is crucial for the stability and accumulation of PINK1 on the outer mitochondrial membrane. Subsequently, PINK1 acts as a signal for Parkin translocation from the cytoplasm to damaged mitochondria, initiating mitophagy [142]. Hirota et al. [143] found that knockout of MAPK1 and MAPK14 or the use of inhibitory drugs significantly inhibited mitophagy induced by starvation and hypoxia. Conversely, another study confirmed the activating effect of AMPK on mitophagy, demonstrating that agonists of the AMPK pathway can stimulate mitophagy, while the MAPK pathway does not affect general macroautophagy [144]. This suggests MAPK exerts specific regulatory roles in mitophagy, with its mechanisms leaning toward stress response and signal amplification, whereas AMPK focuses more on sensing and regulating cellular energy states.

In summary, within the MAPK family, mitophagy can be activated through the ERK1/2, JNK MAPK, and p38/MAPK/Nrf2 signaling pathways. This activation reduces ROS, aids in establishing a neuroprotective barrier, and decreases cell death following cerebral I/R injury. In cerebral I/R injury, MAPK primarily functions as a stress-response-type regulator of mitophagy. By rapidly responding to stress signals to coordinate mitochondrial quality control, it exerts neuroprotective effects.

### 4.4. NF-κB

Nuclear factor-kappa B (NF-κB) serves as a crucial intersection for inflammation, oxidative stress, and mitophagy. This multi-subunit transcription factor is primarily composed of various subunits, including RelA (p65), c-Rel, NF-κB1 (p105/P50), NF-κB2 (p52/p100), and RelB [145]. These subunits share a conserved Rel homology domain (RHD), which is responsible for dimerization, sequence-specific DNA binding, and transcriptional regulation. Traditionally, NF-κB has been considered to play a pro-inflammatory role in cerebral I/R injury. However, a substantial body of evidence indicates that excessive ROS can also activate NF-κB, leading to its involvement in inflammation, immune responses, and acute-phase reactions [146].

Furthermore, A significant aspect of NF-κB’s role is its influence on mitophagy through the expression of the autophagy receptor p62 (also known as SQSTM1), studies by Zhong et al. [147] have demonstrated that NF-κB drives the expression of the autophagy receptor p62/SQSTM1, thereby mediating the clearance of damaged mitochondria: Following mitochondrial injury, Parkin mediates their ubiquitination. p62 recognizes the ubiquitin signal via its ubiquitin-binding domain (UBA) domain and utilizes its LIR domain to target mitochondria to autophagosomes, effectively promoting the clearance of damaged mitochondria. This p62-dependent mitophagy is a key mechanism for regulating inflammatory responses and maintaining cellular homeostasis.

Notably, aberrant activation patterns of NF-κB in the regulation of mitophagy exist under pathological conditions. Research by Li et al. [148] on resveratrol (Res) further elucidated the regulatory link between NF-κB and mitophagy. This natural compound can inhibit the NF-κB pathway (specifically the p50 subunit), thereby downregulating the expression of key mitophagy-related proteins, including PINK1/Parkin, and effectively suppressing excessive mitophagy. This study suggests that abnormal NF-κB activation may lead to imbalanced mitophagy and provides new insights for the potential application of resveratrol in NF-κB-related diseases and subsequent mechanistic studies. This finding further opens new avenues for therapeutic strategies related to NF-κB-related diseases and highlighting resveratrol’s potential for mechanistic studies.

In conclusion, NF-κB’s functions are intricately linked not only to oxidative stress but also to the initiation of p62-dependent mitophagy and potential transcriptional regulation of the PINK1/Parkin pathway. This complex interplay demonstrates that NF-κB’s regulatory effects are highly dependent on the pathological context, expanding its functional scope in cellular quality control.

### 4.5. PI3K/AKT/mTOR

The PI3K/AKT/mTOR signaling pathway is a central regulator of cell survival and metabolism, playing a critical neuroprotective role in cerebral I/R injury [149]. This neuroprotection is mediated through a dual mechanism that coordinates the suppression of excessive mitophagy and the activation of endogenous antioxidant defenses.

On one hand, the pathway suppresses excessive mitophagy by activating mTOR. In the early stages of cerebral I/R injury, damaged neurons release neurotrophic factors such as brain-derived neurotrophic factor (BDNF) and insulin-like growth factor 1 (IGF-1). These factors bind to receptor tyrosine kinases (RTKs) on the cell membrane, leading to the phosphorylation of PI3K. Activated PI3K catalyzes the conversion of phosphatidylinositol(3,4)-bisphosphate (PIP_2_) on the cell membrane to phosphatidylinositol (3,4,5)-trisphosphate (PIP_3_), consequently recruiting and activating downstream AKT [150]. Activated AKT then phosphorylates and inhibits the negative regulatory complex TSC1/TSC2 of mTOR, thereby relieving the inhibition on mTORC1 and activating the mTORC1 signaling pathway [151,152,153].

As a key negative regulator of autophagy, mTOR activation during the acute phase of cerebral ischemia may suppress mitophagy, maintaining mitochondrial functional homeostasis [154]. However, moderate mitophagy can aid in neuronal recovery after cerebral I/R, while excessive mitophagy may lead to energy depletion and exacerbate neuronal damage. Experimental studies have shown that modulating this pathway influences the expression of autophagy-related proteins such as LC3B-II and p62: for instance, Apelin-13 activates the pathway and reduces LC3B-II while increasing p62 accumulation, whereas Piperine inhibits it (with increased PI3K expression but decreased p-AKT/p-mTOR phosphorylation), leading to downregulated Beclin1 and LC3B-II [155,156]. Therefore, regulating the PI3K/AKT/mTOR pathway can help inhibit excessive mitophagy induced by cerebral I/R injury.

On the other hand, the pathway enhances cellular antioxidant defenses through AKT. Studies have shown that activated AKT enhances the phosphorylation of mTOR, which promotes the synthesis and stabilization of HIF-1α, a crucial transcription factor that drives the transcription and expression of the antioxidant gene HO-1. HO-1 catalyzes heme degradation, generating cytoprotective products with antioxidant and anti-inflammatory properties [157]. Furthermore, AKT promotes the dissociation and nuclear translocation of the transcription factor Nrf2, which binds to ARE and upregulates the expression of antioxidant genes including HO-1 and enhancing the cell’s antioxidant defense capabilities [158,159].

In conclusion, the PI3K/AKT/mTOR pathway exerts neuroprotective effects in cerebral I/R injury through a dual mechanism: first, by activating mTOR to inhibit excessive mitophagy and maintain mitochondrial homeostasis; second, by promoting the activation of Nrf2 and HIF-1α via AKT, thereby enhancing the expression of antioxidant genes including HO-1, collectively counteracting cellular damage induced by ischemia–reperfusion.

### 4.6. miR-9-5p

A growing body of evidence highlights the crucial involvement of microRNAs (miRNAs) in the pathogenesis of cerebral I/R injury. Among the numerous miRNAs identified, miR-9-5p stands out as a representative regulator that bridges oxidative stress and mitophagy. This highly conserved, neuron-enriched non-coding RNA has attracted considerable attention due to its central role in modulating redox balance and mitochondrial homeostasis [160,161]. Recent studies have revealed its central role in modulating oxidative stress and mitochondrial function in neurodegenerative diseases and brain injury models. In cerebral I/R models, the expression level of miR-9-5p is altered, and its dysregulation is closely associated with neuronal apoptosis, aberrant autophagy, and inflammatory responses [162]. Notably, miR-9-5p expression is significantly downregulated in hypoxic–ischemic (HI) models and stroke patients, whereas experimental overexpression of miR-9-5p reduces cerebral infarct volume and improves neurological function [163]. Evidence indicates that miR-9-5p exerts its protective effects by targeting multiple pathways. It can inhibit the transcriptional activity of ZBTB20, which weakens the interaction between Nrf2 and its inhibitor Keap1. This facilitates the nuclear translocation of Nrf2, activating the expression of the antioxidant enzyme SOD2 and ultimately alleviating oxidative stress [164]. Building on this, its role extends to regulating the PI3K/AKT/mTOR pathway. Specifically, Nrf2, upon activation by AKT, translocates to the nucleus to upregulate antioxidant genes like HO-1. This demonstrates that miR-9-5p’s role focuses on the critical protective hub—especially the Nrf2-mediated antioxidant response—highlighting its core position in coordinating a multi-target network against cerebral I/R injury.

Supporting these findings, recent cellular experiments demonstrate that miR-9-5p ameliorates mitochondrial structural damage, reduces autophagosome formation, downregulates LC3-I/II conversion, and upregulates Parkin protein expression, thereby suppressing excessive mitophagy and alleviating oxidative stress in ischemic stroke [165].

In summary, miR-9-5p plays a multifaceted protective role in CIRI, primarily through regulating the ZBTB20/Nrf2/Keap1/SOD2 axis, GSK-3β, and mitophagy-related pathways. Key characteristics of miR-9-5p include its specificity for the nervous system and its function as an upstream regulatory factor. collectively conferring antioxidant effects, improving mitochondrial function, and inhibiting aberrant mitophagy. Additionally, miR-9-5p’s potential as a biomarker and therapeutic target for cerebral I/R injury warrants further exploration.

### 4.7. SIRT

Silent Information Regulator 1 (SIRT1) is a NAD^+^-dependent deacetylase that regulates levels of ROS and RNS, oxidative stress responses, and the activity of antioxidant enzymes (such as SOD, CAT, and GPx) through the deacetylation of histone and non-histone targets (including Nrf2, FOXO3, NF-κB, and p53) [166,167]. The SIRT family consists of seven members (SIRT1-SIRT7), with SIRT1 being one of the most widely studied and functionally diverse members, confirmed to have neuroprotective effects in cerebral ischemia, reducing neuronal death and alleviating oxidative stress and neuroinflammatory responses [168,169,170].

Following cerebral I/R, cells experience an energy crisis that leads to significant increases in the AMP/ATP ratio and NAD^+^/NADH ratio. Since NAD^+^ is an essential cofactor for the deacetylase activity of SIRT1, the elevation of NAD^+^ levels activates the deacetylation function of SIRT1 [171,172]. Under conditions of cerebral I/R, SIRT1 acts as a central regulator by activating two key pathways.

#### 4.7.1. SIRT1–FOXO3

Particularly under conditions of oxidative stress, activated SIRT1 can directly deacetylate forkhead box O3 (FOXO3) by removing acetyl groups from specific lysine residues (such as K242, K245, and K259), thereby relieving FOXO3 from its inactive state, enhancing its transcriptional activity, and facilitating its translocation to the nucleus to bind to target gene promoters [173,174]. Deacetylated FOXO3 transcription factor can upregulate the expression of antioxidant genes [175].

In recent years, FOXO3 has also been found to directly bind to the upstream promoter region of the BNIP3 gene, promoting BNIP3 transcription and protein expression. BNIP3 then maintains the stability of PINK1 at the outer mitochondrial membrane, assisting in the recruitment of Parkin, and ultimately activating the PINK1/Parkin-mediated mitophagy pathway to selectively remove damaged mitochondria [176,177].

#### 4.7.2. SIRT1–PGC-1α

Moreover, activated SIRT1 can also enhance cellular antioxidant capacity and promote mitochondrial biogenesis by deacetylating peroxisome proliferator-activated receptor γ coactivator 1-alpha (PGC-1α). On one hand, PGC-1α mitigates oxidative damage and lipid peroxidation by enhancing overall antioxidant defenses, while simultaneously suppressing NLRP3 inflammasome activation and the release of proinflammatory cytokines such as IL-1β and TNF-α, thereby reducing neuroinflammatory responses [178,179]. On the other hand, the SIRT1-PGC-1α axis restores mitochondrial homeostasis by promoting mitochondrial biogenesis and dynamic remodeling, as evidenced by the upregulation of OPA1 expression and inhibition of excessive activation of DRP1, which reduces mitochondrial fragmentation [180].

Additionally, phosphorylation of AMPK enhances the transcriptional coactivator capability of PGC-1α and forms a positive feedback loop of AMPK-SIRT1-PGC-1α by elevating NAD^+^ levels [178,181].

#### 4.7.3. SIRT3

Unlike SIRT1, which primarily orchestrates adaptive transcriptional responses in the nucleus and cytoplasm, the mitochondrial NAD^+^-dependent deacetylase SIRT3 provides a localized regulatory node within mitochondria during cerebral I/R. It directly reshapes mitochondrial redox homeostasis and quality control. In neuronal oxygen-glucose deprivation/reperfusion (OGD/R) models, SIRT3 expression declines during the reperfusion phase. Pharmacological activation of SIRT3 attenuates neuronal injury, suppresses excessive ROS production, and preserves mitochondrial integrity. Collectively, these findings underscore the therapeutic relevance of targeting the mitochondrial deacetylase signaling pathway during the reperfusion window [182].

Mechanistically, SIRT3 constrains mitochondrial oxidative stress mainly through deacetylation-dependent enhancement of mitochondrial antioxidant defenses. For example, in diabetic cerebral I/R models, melatonin activates the Akt–SIRT3–SOD2 axis and reduces SOD2 acetylation, whereas SIRT3 inhibitor or SIRT3 silencing largely abolishes these protective effects [183]. Simultaneously, SIRT3 promotes post-ischemic mitochondrial structural repair and functional recovery by enhancing OPA1 expression, thereby facilitating mitochondrial fusion and improving mitochondrial energy metabolism. These mechanisms collectively mitigate oxidative stress and enhance neurological function [184].

In recent years, SIRT3 has also been implicated in mitophagy associated quality control during cerebral I/R. To perform this role, SIRT3 supports selective clearance of dysfunctional mitochondria by promoting PINK1 stability and Parkin recruitment, thereby facilitating PINK1/Parkin dependent mitophagy to eliminate damaged mitochondria and limit secondary amplification of ROS during reperfusion [185]. Concurrently, SIRT3 achieves the goal of establishing a mitochondrial environment capable of autophagy by coordinating mitochondrial homeostasis programs, such as maintaining mitochondrial proteome integrity through activation of the mitochondrial unfolded protein response (UPR^mt^), thereby enhancing overall mitochondrial recovery capacity following transient ischemia [186]. Notably, the SIRT3 module may synergize with upstream NAD^+^-sirtuin networks. Under cerebral I/R stress, SIRT1 enhances SIRT3 activity through deacetylation, establishing functional nuclear-mitochondrial signaling coupling. This mechanism redirects the dynamic equilibrium between mitophagy and oxidative stress from a vicious cycle of injury amplification toward a virtuous trajectory of repair and reconstruction, ultimately promoting the restoration of mitochondrial structure and function [187].

In summary, the sirtuin network constitutes a critical NAD^+^-dependent regulatory axis in cerebral I/R injury. SIRT1 functions as a central metabolic–redox integrator by deacetylating FOXO3 and PGC-1α, thereby enhancing antioxidant defense, promoting PINK1/Parkin-mediated mitophagy, stimulating mitochondrial biogenesis, and suppressing neuroinflammation. In parallel, SIRT3 reinforces local redox homeostasis and quality control by activating SOD2, facilitating mitochondrial fusion, and supporting mitophagy and UPR^mt^ signaling. Together, coordinated SIRT1–SIRT3 signaling reestablishes mitochondrial integrity and limits oxidative stress, ultimately promoting neurological recovery after cerebral I/R.

The intricate crosstalk between oxidative stress and mitophagy underscores the significance of precise regulatory mechanisms in orchestrating a protective versus damaging response in cerebral I/R injury. From AMPK/ULK1 and Cardiolipin (CL), which sense energy and oxidative stress, to MAPK and NF-κB, which integrate stress and inflammatory signals, and further to PI3K/AKT/mTOR, miR-9-5p, SIRT1 and SIRT3, which coordinate survival and metabolism—these pathways intersect to form a delicate equilibrium that collectively determines cellular fate. Deepening our understanding of the spatiotemporal activation patterns and interactions within this network will provide a critical therapeutic window for developing novel neuroprotective strategies targeting mitochondrial quality control.

## 5. Treatment Strategy

Therapeutic interventions for ischemic stroke primarily revolve around three major strategies: First, targeting mitophagy—moderately activating mitophagy to enhance mitochondrial quality control, thereby protecting neurons from injury, while simultaneously suppressing excessive mitophagy to prevent exacerbating neural damage. Second, targeting oxidative stress by utilizing endogenous and exogenous antioxidants to neutralize ROS and mitigate oxidative damage. Building upon this foundation, dual-target intervention strategies simultaneously modulate mitophagy and counteract oxidative stress, leveraging their interdependent pathways to synergistically enhance therapeutic efficacy.

### 5.1. Therapeutic Strategies Targeting Mitophagy

#### 5.1.1. Activating Moderate Mitophagy

Studies have demonstrated that activating moderate mitophagy specifically during the early phase of reperfusion exerts neuroprotective effects and can positively influence the prognosis of ischemic stroke. It should be noted that, moderate mitophagy is best characterized by the balance it strikes in cellular homeostasis. Key signaling pathways involved in activating mitophagy include PINK1/Parkin, BNIP3, and FUNDC1, among others. Numerous studies have identified various therapeutic approaches, such as Ligustilide [188], electroacupuncture [189], and metformin [190] that can activate mitophagy in the early reperfusion stage via the PINK1/Parkin pathway, thereby improving mitochondrial function and alleviating neuronal damage. Additionally, rapamycin, a well-established inducer of mitophagy and an inhibitor of mTOR, promotes mitophagy primarily by suppressing mTOR activity, which enhances neuronal survival and reduces cell death following I/R injury [191]. However, rapamycin is associated with systemic adverse effects [192], which may limit its translational applicability in acute ischemic stroke. Moreover, to overcome the limitations of first generation rapalogs, next generation mTOR targeting agents, such as rapamycin analogs (e.g., everolimus, temsirolimus) and ATP competitive mTOR kinase inhibitors with broader mTORC1/2 inhibition, have been developed and are being actively evaluated in clinical settings [193]; nonetheless, their efficacy, CNS penetrance, and safety profile in cerebral I/R still require dedicated validation.

Urolithin A (UA) has been shown to reduce infarct volume and protect neurological function after cerebral ischemia in the middle cerebral artery occlusion (MCAO) model [194]. This process not only enhances mitochondrial quality control but also facilitates the clearance of damaged mitochondria by upregulating BNIP3 and FUNDC1, thereby helping to maintain cellular physiological function and energy homeostasis.

In summary, activating moderate mitophagy through pharmacological agents (such as UA and rapamycin) or non-pharmacological means (such as EA), particularly during the early phase of reperfusion, has emerged as an effective neuroprotective strategy. However, a critical challenge lies in defining the appropriate level of “moderation” and achieving precise spatiotemporal regulation. Future efforts should focus on fine-tuning mitophagic activity rather than merely enhancing it, in order to avoid shifting toward detrimental excessive mitophagy. The current evidence in this field primarily stems from basic research, and its potential for clinical application awaits rigorous validation through prospective clinical trials.

#### 5.1.2. Inhibiting Excessive Mitophagy

Although mitophagy plays a crucial role in eliminating damaged mitochondria and maintaining cellular homeostasis, it is not always beneficial, as excessive activation of mitophagy may lead to the degradation of essential cellular components, thereby exacerbating injury [195]. Evidence indicates that inhibiting the overactivation of mitophagy can mitigate neuronal apoptosis in the brain [196]. Therefore, precisely regulating the extent of mitophagy to prevent its excessive activation emerges as an important therapeutic strategy.

Recent studies have identified various compounds that can inhibit excessive mitophagy by suppressing signaling pathways and directly regulating mitochondrial function, thereby exerting neuroprotective effects. In terms of signal pathway inhibition, artesunate can suppress FUNDC1-mediated excessive mitophagy by activating the AMPK/mTOR/TFEB pathway, reducing cerebral I/R injury [56]. Oridonin (Ori) exhibits effects similar to mitophagy inhibitors such as 3-MA, by downregulating the AMPK mitophagy signaling pathway, thereby decreasing excessive mitophagy and caspase-9-dependent neuronal apoptosis [196]. Piperine (PIP), the primary active component of black pepper, has also been shown to confer neuroprotection by inhibiting the PI3K/AKT/mTOR signaling pathway and reducing excessive mitophagy [156].

In terms of direct regulation of mitochondrial function, dexmedetomidine (DEX), a highly selective α2 adrenergic receptor agonist commonly used for sedation and analgesia in clinical settings, has been demonstrated by Tang et al. [197] to reduce excessive mitophagy in cerebral I/R injury by inhibiting mitochondrial calcium uniporter (MCU), thereby facilitating recovery from cerebral I/R injury. It is noteworthy that the existing clinical application foundation of dexmedetomidine provides potential advantages for its translation into the treatment of neurological disorders.

It is noteworthy that strategies to inhibit excessive mitophagy are diverse; artesunate and piperine function through modulation of the upstream mTOR pathway, while dexmedetomidine directly targets mitochondrial calcium homeostasis. This multi-target characteristic suggests that excessive mitophagy may be triggered by various upstream stress signals, necessitating the selection of personalized inhibition strategies based on the specific stages and characteristics of the injury.

The various therapeutic strategies targeting mitophagy and their mechanisms of action and effects are summarized in Table 2.

### 5.2. Therapeutic Strategies Targeting Oxidative Stress

Excessive oxidative stress is a central component of I/R injury. In response, therapeutic strategies primarily fall into two categories: enhancing endogenous antioxidant systems and supplementing exogenous antioxidants. With hundreds of antioxidant compounds identified to date, a comprehensive review is beyond our scope. To navigate this complexity, we have selected representative examples based on a set of targeted criteria. Accordingly, preference was given to agents with: (1) evidence supporting neuroprotection in cerebral I/R models and/or clinical ischemic stroke settings; (2) mechanistic representativeness, covering both direct ROS scavenging and indirect reinforcement of endogenous defenses; and (3) translational relevance, including pharmacological feasibility and safety/drug–drug interaction profiles. These approaches aim to directly or indirectly neutralize excessive ROS and restore redox balance. However, traditional antioxidant strategies typically rely on non-targeted ROS scavenging, which may fail to adequately address the primary source—mitochondrial ROS production—thereby limiting therapeutic efficacy. This underscores the need for more precise organelle-targeted intervention strategies.

#### 5.2.1. Endogenous Antioxidants

CoQ10 is the only fat-soluble antioxidant that can be synthesized de novo by human cells. It not only directly scavenges ROS and reduces the level of MDA, a marker of lipid peroxidation, but also indirectly enhances the activity of other endogenous antioxidants, such as GSH, GPx, and SOD, thereby collectively exerting antioxidative stress effects [198,199]. Studies have shown that the combination of CoQ10 and metformin has a better antioxidant effect in post-stroke treatment than either agent alone [200]. However, when used concomitantly with warfarin, CoQ10 may reduce the anticoagulant effect, and its combination with vitamin K antagonists can increase the risk of bleeding [201]. Nevertheless, the clinical efficacy of short-term CoQ10 administration in acute cerebral ischemia has not reached the significant level observed in experimental studies [202], which may be related to challenges in translating experimental findings into clinical practice. This discrepancy highlights the limitations of non-targeted ROS scavenging strategies, underscoring the urgent need to focus on mitochondrial-targeted approaches that address ROS production at its source. Future clinical trials with larger sample sizes, longer durations, and higher doses are needed to further clarify its therapeutic effects.

#### 5.2.2. Exogenous Antioxidants

The antioxidant effects of natural products have garnered increasing attention. For instance, anemonin, baicalein, and Ginkgo biloba extract EGB761 demonstrate the ability to directly scavenge ROS. Moreover, anemonin can restore the activity of endogenous antioxidant enzymes—such as SOD, CAT, GSH, and GPx—while inhibiting lipid peroxidation, thereby preserving cell membrane integrity and reversing oxidative stress imbalance induced by cerebral I/R injury [203]. Baicalein not only reduces lipid peroxidation-mediated oxidative stress by inhibiting 12/15-lipoxygenase (12/15-LOX) but also exerts antioxidant effects by downregulating the AMPK/Nrf2 pathway [204,205]. Isorhamnetin, a constituent of the Ginkgo biloba extract EGB761, has been shown to activate antioxidant defenses via the Keap1-Nrf2-ARE signaling pathway [206]. Further studies indicate that EGB761 also exerts neuroprotective effects through the Akt-CREB-BDNF pathway, suggesting its potential value in the treatment of neurological disorders [207]. However, the effects of these compounds on mitochondrial ROS production or mitochondrial antioxidant systems remain less explored, indicating a potential area for future investigation.

In addition, the compound LFHP-1c, a novel inhibitor of phosphoglycerate mutase family member 5 (PGAM5), directly binds to and inhibits PGAM5, relieving its suppression of Nrf2. This promotes Nrf2 nuclear translocation and upregulates the expression of antioxidant genes such as HO-1 and NAD(P)H quinone dehydrogenase 1 (NQO1), thereby reducing ROS accumulation [208]. Similarly, Li et al. reported that urolithin B (UB) enhances the expression and nuclear translocation of Nrf2 and HO-1, strengthening cellular antioxidant capacity and further confirming the critical role of the Nrf2/HO-1 signaling pathway in antioxidant mechanisms [209].

Edaravone, a clinically used free radical scavenger, has shown therapeutic potential in acute ischemic stroke (AIS) and amyotrophic lateral sclerosis (ALS) [210,211]. Beyond directly neutralizing various free radicals, it protects astrocytes from oxidative stress by modulating the Akt/Bcl-2/Caspase-3 signaling axis and mitigates inflammatory damage by suppressing pro-inflammatory cytokine expression [212]. Studies have shown that co-administration of edaravone during t-PA infusion significantly improves early recanalization rates and promotes neurological recovery without increasing the risk of hemorrhagic transformation [213]. Consistent results have been observed in experimental models [214]. This highlights that the timing of administration and the specific targeting of oxidative stress pathways could greatly influence the outcomes of antioxidant therapies.

Therefore, we hypothesize that the combination of edaravone and tPA may produce synergistic therapeutic effects in clinical practice.

The various therapeutic strategies targeting oxidative stress and their mechanisms of action and effects are summarized in Table 3.

### 5.3. Therapeutic Strategies Targeting Dual Targets

Given the complex pathophysiology of cerebral I/R injury involving multiple factors, therapeutic strategies targeting dual or multiple pathways are gaining increasing attention to more precisely address its intricate pathological processes. This multifaceted approach not only counters the oxidative stress and mitophagy commonly observed in cerebral I/R injury but also promotes the clearance of damaged cellular components, thereby enhancing cellular resilience against ischemic injury.

#### 5.3.1. Mitochondrial Antioxidants

Mounting evidence suggests that targeting mitophagy offers superior advantages over direct ROS scavenging in mitigating excessive ROS, as the latter approach may concurrently suppress critical autophagic signaling pathways such as mTOR or AMPK. From the perspectives of safety and efficacy in countering oxidative stress, mitochondrial antioxidants have also garnered significant attention. These compounds primarily function by restoring normal mitochondrial activity, thereby reducing ROS generation and alleviating oxidative damage.

UBIAD1, a key enzyme involved in CoQ10 biosynthesis localized to both mitochondria and the Golgi apparatus, exhibits antioxidant properties in melanoma studies by synthesizing CoQ10 and inhibiting lipid peroxidation [215]. Huang et al. [216] demonstrated that UBIAD1 overexpression restores mitochondrial function following cerebral I/R injury, enhances the activity of mitochondrial respiratory chain complexes, elevates levels of antioxidants such as SOD and GSH, and suppresses the production of ROS and MDA, thereby attenuating oxidative stress.

Methylene blue (MB), an oxygen-reducing dye with high lipophilicity, readily crosses the BBB and rapidly accumulates in the central nervous system [217]. Previous studies have reported the neuroprotective effects of MB after cerebral I/R injury [218,219]. Lu et al. [220] found that MB treatment markedly ameliorates mitochondrial dysfunction, reduces mitochondrial ROS generation, preserves mitochondrial membrane potential, and prevents apoptosis, thereby protecting neurons from oxidative damage. Additional experiments confirmed that low-dose MB can directly transfer electrons to cytochrome c oxidase in the mitochondrial electron transport chain, promoting ATP synthesis while minimizing the generation of harmful byproducts such as superoxide radicals. Moreover, MB inhibits the overexpression of inducible nitric oxide synthase (NOS), thereby reducing inflammation-associated oxidative stress [221].

#### 5.3.2. SIRT1 Agonist

Resveratrol, a recognized SIRT1 agonist, primarily enhances mitochondrial function and promotes mitophagy through activation of the SIRT1-mediated AMPK/PGC-1α pathway [222]. The three hydroxyl groups (·OH) in its structure can react with free radicals, neutralizing ROS and other reactive species via electron or hydrogen atom transfer, thereby alleviating oxidative stress [223]. Additional studies have confirmed that resveratrol exerts both antioxidant and anti-apoptotic effects [224,225].

In the regulation of mitochondrial homeostasis and oxidative stress, curcumin exhibits multi-target characteristics similar to resveratrol but operates through distinct pathways. Curcumin upregulates SIRT1 expression, enhances mitochondrial membrane potential, and improves the activity of mitochondrial complexes, thereby ameliorating mitochondrial dysfunction induced by ischemic stroke—effects that can be reversed by the SIRT1 inhibitor Sirtinol. Moreover, curcumin activates the AMPK/PINK1/Parkin signaling pathway to facilitate the selective clearance of damaged mitochondria and maintain mitochondrial homeostasis [226]. It also binds to the mitochondrial succinate dehydrogenase (specifically the SDHC subunit), modulating its activity to induce moderate ROS production, which in turn triggers protective mitophagy [227].

Furthermore, the molecular structure of curcumin confers antioxidant capacity, enabling it to reduce mitochondrial ROS generation and mitigate oxidative stress. Concurrently, curcumin suppresses neuroinflammation by decreasing TNF-α levels in brain tissue, thereby reducing inflammation-associated oxidative damage [228].

#### 5.3.3. AMPK Agonists

Studies have shown that major ginsenoside components, such as Rd and Compound K, act as key regulators of the AMPK/mTOR pathway. Through this central mechanism, they initiate moderate mitophagy to eliminate dysfunctional mitochondria and maintain intracellular energy homeostasis [229].

In countering oxidative stress, ginsenosides operate through a particularly systematic mechanism: they specifically activate the Nrf2/ARE pathway—a central endogenous antioxidant signaling axis—thereby upregulating the expression of antioxidant proteins including HO-1 and NQO-1 [230]. Simultaneously, other ginsenoside constituents, such as Rb1 and Re, also serve as direct antioxidants [231,232,233,234]. These interconnected mechanisms collectively form the foundation for the potent antioxidant and mitophagy-modulating activities of ginsenosides, providing a mechanistic basis for their neuroprotective effects in ischemic stroke.

#### 5.3.4. PI3K-Akt Pathway Agonists

Puerarin, a major extract from the roots of Pueraria lobata, acts as an agonist of the PI3K-Akt pathway. Experimental studies have demonstrated that puerarin exerts multi-faceted protective effects against cerebral I/R injury. It not only improves cardio-cerebrovascular circulation but also enhances ischemic tolerance by increasing superoxide dismutase activity and suppressing free radical generation, thereby attenuating ischemic reperfusion injury.

Research by Zhang et al. [235] revealed that puerarin alleviates cerebral I/R injury both in vivo and in vitro by activating the PI3K/Akt/Nrf2 signaling pathway, which suppresses oxidative stress and neuronal apoptosis.

The various therapeutic strategies targeting dual targets and their mechanisms of action and effects are summarized in Table 4.

## 6. Discussion

Cerebral I/R injury is a key pathological process leading to the deterioration of neurological function after ischemic stroke, and its mechanism involves complex crosstalk among various cellular stress responses. Notably, a dual-target therapeutic strategy that simultaneously modulates mitophagy and oxidative stress is more aligned with the multifaceted pathology of cerebral I/R injury. Distinguishing itself from reviews focusing on isolated mechanisms, this review systematically integrates the bidirectional regulatory network between oxidative stress and mitophagy, elucidating how their dynamic interplay dictates the progression of injury or repair. This review is dedicated to dissecting this critical bidirectional relationship.

Mitophagy, as an important quality control system, can remove damaged mitochondria and maintain intracellular homeostasis; while the reactive oxygen burst induced after cerebral I/R will cause oxidative stress and damage to macromolecules, and activate cell death signals. These two factors do not exist independently but form a finely regulated interactive network: moderate oxidative stress can activate protective mitophagy through pathways such as AMPK/ULK1 and cardiolipin exocytosis, and normal mitophagy can remove the source of ROS and inhibit further oxidative damage, forming a negative feedback loop. Once out of balance, excessive oxidative stress will induce abnormal mitophagy, which in turn further aggravates ROS accumulation, forming a vicious cycle.

The regulation of this interaction network holds therapeutic potential, including strategies such as regulating mitophagy and combining the use of antioxidants for multi-target intervention. However, this field still faces many challenges: (1) Lack of quantitative benchmarks defining “moderate” or “protective” levels of mitophagy; (2) Inherent differences between animal models and human pathophysiology; (3) Dual BBB permeability and systemic drug safety. These limitations highlight a critical translational gap: clear mechanistic understanding does not equate to clinical feasibility. Most interventions remain confined to the preclinical stage, constrained by these practical obstacles.

In conclusion, a thorough understanding of the mitophagy–oxidative stress interplay, therefore, offers a promising avenue for alleviating cerebral I/R injury and improving stroke prognosis. To bridge the existing translational gaps, future research should specifically focus on multi-target combined regulation to synchronously restore mitophagy and redox balance; temporally sequential intervention strategies that address different injury phases; as well as personalized therapeutic windows based on disease progression and patient-specific biomarkers. Ultimately, advancing these strategic directions is crucial to accelerate the development of effective therapies with significant clinical implications.

## Figures and Tables

**Table 1:** The cell type specific characteristics of mitophagy in cerebral I/R injury.

Brain Cell Type	Upstream Regulators	Cell Specific Functional Consequences	Source
Neurons	Energy depletion; Ca^2+^ overload; ROS accumulation; PINK1/Parkin; BNIP3/NIX	Determines neuronal survival threshold; moderate activation reduces oxidative damage and apoptosis; excessive activation aggravates ATP depletion and cell death	Li, et al. (2023) [9] Wu, et al. (2021) [57]
Astrocytes	Metabolic stress; hypoxia signaling; oxidative stress	Maintains metabolic homeostasis; supports neurons; regulates redox and inflammation; provides mitochondrial support	Cao, et al. (2021) [58] Zhang, et al. (2024) [59]
Microglia	mtROS accumulation; mitochondrial DNA release; NLRP3 inflammasome signaling; PINK1/Parkin	Regulates inflammatory activation; suppresses NLRP3 driven IL-1β; influences polarization and secondary neuronal injury	Lv, et al. (2021) [60] Song, et al. (2025) [61]
BMECs	Oxidative stress; mitochondrial dysfunction; AMPK metabolic stress signaling	Preserves BBB integrity; stabilizes tight junctions; reduces vascular permeability and edema	Guo, et al. (2023) [62] Tang, et al. (2025) [63] Wang, et al. (2023) [64]

**Table 2:** Therapeutic Strategies Targeting Mitophagy.

Treatment Method	Pathway	Effect	Source
Ligustilide	Activate Pink1/Parkin	Promote moderate mitophagy	Mao, et al. (2022) [188]
Electroacupuncture	Activate Pink1/Parkin	Promote moderate mitophagy	Wang, et al. (2019) [189]
Metformin	Activate Pink1/Parkin	Promote moderate mitophagy	Guo, et al. (2023) [190]
Urolithin A (UA)	Activate Pink1/Parkin	Promote moderate mitophagy	Lin, et al. (2020) [194]
Rapamycin	Inhibit mTOR	Promote moderate mitophagy	Li, et al. (2014) [191]
Artesunate	Inhibit FUNDC1	Reduce excessive mitophagy	Wang, et al. (2025) [56]
Oridonin (Ori)	Inhibit AMPK	Reduce excessive mitophagy	Li, et al. (2023) [196]
Piperine (PIP)	Inhibit PI3K/AKT/mTOR	Reduce excessive mitophagy	Zhang, et al. (2022) [156]
Dexmedetomidine (DEX)	Inhibit mitochondrial calcium uniporter (MCU)	Reduce excessive mitophagy	Tang, et al. (2019) [197]

**Table 3:** Therapeutic Strategies Targeting Oxidative Stress.

Treatment Method		Pathway	Effect	Source
Endogenous antioxidants	CoQ10	Directly eliminate ROS; indirectly enhance GSH, GPx and SOD	Reduce oxidative stress damage	Xie, et al. (2020) [198]
Exogenous antioxidants	anemonin	Directly eliminate ROS, and restore the activities of SOD, CAT, GSH, and GPx	Reduce oxidative stress damage	Jia, et al. (2014) [203]
	Baicalein	Directly eliminate ROS, inhibit 12/15-LOX, AMPK/Nrf2	Reduce oxidative stress damage	van Leyen, et al. (2006) [204] Yuan, et al. (2020) [205]
	ginkgo biloba extract EGB761	Directly eliminate ROS and activate Keap1-Nrf2-ARE	Reduce oxidative stress damage	Liu, et al. (2007) [206] Zhang, et al. (2012) [207]
	LFHP-1c	Inhibit PGAM5 and activate Nrf2/HO-1	Reduce oxidative stress damage	Gao, et al. (2021) [208]
	Urolithin B(UB)	Activate Nrf2/HO-1	Reduce oxidative stress damage	Li, et al. (2024) [209]
	Edaravone	Directly eliminate free radicals and activate Akt/Bcl-2/Caspase-3	Reduce oxidative stress damage	Guo, et al. (2020) [212]

**Table 4:** Therapeutic Strategies Targeting Dual Targets.

	Treatment Method	Pathway	Effect	Source
Mitochondria-Targeted Antioxidant	UBIAD1	Synthetic Coenzyme Q10	Increase SOD and GSH, and inhibit the generation of ROS and MDA	Arslanbaeva, et al. (2022) [215] Huang, et al. (2022) [216]
	MB	Acts on cytochrome C oxidase; inhibits the excessive expression of inducible NOS	Reduce mitochondrial ROS production; reduce inflammatory-related oxidative stress damage	Lu, et al.(2016) [220] Rodriguez, et al. (2016) [221]
SIRT1 agonist	Resveratrol	Activate the AMPK/PGC-1α	Improve mitochondrial function, promote mitophagy; reduce oxidative stress	He, et al. (2017) [222] Oh, et al. (2017) [223]
	Curcumin	Activate the AMPK/PINK1/Parkin; Combine SDHC; Reduce TNF-α	Maintain mitochondrial homeostasis; reduce ROS; inflammatory-related oxidative stress damage	Jin, et al. (2022) [226] Miao, et al. (2016) [228]
AMPK agonist	Ginsenosides	Regulate the AMPK/mTOR pathway; activate Nrf2/ARE	Initiate moderate mitophagy; drive HO-1 and NQO-1	Guo, et al. (2014) [229] Fernández-Moriano, et al. (2017) [230] Zhao, et al. (2022) [231]
PI3K-Akt pathway agonist	Puerarin	Activate the PI3K/Akt/Nrf2 and PI3K/Akt/mTOR	Improve the circulation of the heart and blood vessels, inhibit oxidative stress and neuronal apoptosis	Gao, et al. (2022) [232] Chen, et al. (2025) [233] Yuan, et al. (2017) [234] Zhang, et al. (2023) [235]

## Data Availability

No new data were created or analyzed in this study. Data sharing is not applicable to this article.

## Abbreviations

The following abbreviations are used in this manuscript:

I/R	Ischemia/Reperfusion
ROS	Ischemic Stroke
AMPK	AMP-activated protein kinase
ULK1	Unc-51 like autophagy activating kinase 1
Nrf2	nuclear factor erythroid 2-related factor 2
Keap1	Kelch-like ECH-associated protein 1
HIF-1α	hypoxia-inducible factor-1α
PI3K	Phosphatidylinositol 3-kinase
mTOR	mammalian target of rapamycin
NF-κB	Nuclear factor-kappa B
ARE	antioxidant response element
